# Students' Perceptions of School Nurse Services and Their Self‐Reported Health Status in Hungary: A Cross‐Sectional Study

**DOI:** 10.1002/nop2.70670

**Published:** 2026-06-27

**Authors:** Ilona Karácsony, Tímea Csákvári, Annamária Pakai

**Affiliations:** ^1^ Department of Prevention and Perinatal Medicine, Institute of Basics of Health Sciences, Midwifery and Health Visiting, Faculty of Health Sciences University of Pécs Pécs Hungary; ^2^ Department of Health Economics and Health Care Management, Institute of Health Insurance, Faculty of Health Sciences University of Pécs Zalaegerszeg Hungary; ^3^ Institute of Emergency Care, Pedagogy of Health and Nursing Sciences, Faculty of Health Sciences University of Pécs Pécs Hungary

**Keywords:** health knowledge, health perception shaping, health status, satisfaction, school nurse services

## Abstract

**Aim:**

To assess the perceived effectiveness of the school nurse services through students' responses and to examine whether there is a detectable relationship between the students' subjective health status and the school nurse's service delivery.

**Design:**

We conducted our cross‐sectional, quantitative research in the West Transdanubian region of Hungary using convenience sampling among high school students (*n* = 429). Given the non‐probability sampling, the findings may not be generalizable to all student populations.

**Methods:**

Data were collected using standardized (SF‐36, EQ‐VAS, questions on the prevalence of subjective health complaints from the HBSC survey) and a self‐developed questionnaire. Chi‐square tests, correlation analyses and multivariable linear regression analyses were performed using SPSS Statistics 27.0 software.

**Results:**

Students reported a higher level of health‐related knowledge received from the school nurse, along with better perceived applicability of this knowledge in everyday life (*p* < 0.05). Nearly three quarters of respondents indicated that the school nurse's activities contributed to the development of their health‐related attitudes and this perception showed a statistically significant association with both the quantitative and qualitative evaluation of the care provided (*p* < 0.05). Higher ratings of the perceived effectiveness of school nursing activities were associated with more favourable self‐rated mental health and a lower level of reported health complaints; however, these associations were weak in magnitude.

**Conclusion:**

The results revealed statistically significant but weak associations between perceived school nurse services and selected indicators of students' self‐rated health status. Due to the cross‐sectional design and the use of self‐reported data, causal inferences cannot be drawn. Nonetheless, the findings suggest that more favourable quantitative and qualitative evaluations of the care provided are linked to higher perceived effectiveness of school nurse activities. Further research on quality indicators of school nurse services and approaches tailored to students' needs may be warranted.

**Implications for the Profession and/or Patient Care:**

The observed associations between students' perceptions of school nurse services and their health perceptions underscore the importance of continuous professional development for school nurses. This may include advanced training in health education and communication skills to further support the perceived effectiveness of their activities.

**Impact:**

*What problem did the study address?* We addressed the perceived effectiveness of school nurse services from the perspective of students and examined whether there is a detectable relationship between students' subjective health status and the services provided by school nurses. *What were the main findings?* The study found that higher levels of health‐related knowledge reported as being provided by the school nurse were associated with more favourable student ratings of the applicability of this knowledge in everyday life. Students who reported more positive experiences with school nurse activities also tended to report more favourable health perceptions, which were reflected in the indicators used to evaluate the services. In addition, higher perceived effectiveness of school nurse services was associated with more favourable self‐reported mental health and vitality, as well as a lower level of reported health complaints. *Where and on whom will the research have an impact?* The findings may be particularly relevant for school nurses, as they highlight the importance of their role and the potential need for continuous attention to service quality from the students' perspective. They may also be informative for students and school communities by underscoring that health education and services provided by school nurses are associated with more favourable self‐reported health perceptions and overall well‐being.

**Patient or Public Contribution:**

No patients or members of the public were involved in the design, conduct, analysis, or reporting of this study beyond students' participation as anonymous questionnaire respondents.

## 1 Introduction

Schools are key settings for shaping young people's health behaviours and supporting their overall well‐being. Since health‐related habits formed during adolescence often persist into adulthood, the public education system carries substantial responsibility for promoting population health (Török 2015). According to the World Health Organizaton (WHO), schools provide a unique environment for delivering health education, early prevention and targeted support (World Health Organization 2018). Within this system, the school nurse plays an essential role in improving students' health knowledge, identifying emerging issues and contributing to long‐term health development.

## 2 Background

Students' health affects their learning ability and creates opportunities for achieving academic goals. An educational and nurturing organization that supports health enhances learning outcomes and healthier students tend to perform better academically (Somhegyi 2016). Positive experiences may serve as protective factors for health, while negative ones pose risks. The relationship between health and learning underscores the importance of having health professionals in schools and involving them in supporting chronically absent students, promoting student health and creating a school environment that fosters well‐being (Kostenius 2023; Lee et al. 2025; McCabe et al. 2026).

School health service professionals, including school doctors and school nurses, play a key role in strengthening the health and well‐being of children and adolescents through public health and healthcare tasks (Cygan et al. 2020; McCabe et al. 2025; Barrett 2026). For the school‐aged, the professionals within the school environment are involved in health supervision, screening and addressing physical and psychological issues, as well as supporting the development of protective health behaviours through individual counselling and community health promotion. They provide assistance in accessing additional healthcare services as needed, coordinate the care process for children with chronic conditions and make referrals to other services (Schroeder et al. 2018; Hoekstra et al. 2016).

According to Rising Holmström et al., students aged 6–16 (*n* = 734) identify the school nurse as a key person in health promotion and the identification of health problems. The school nurse has crucial responsibilities in maintaining students' health and supporting their learning, including creating a healthy school environment, conducting health screenings and providing advice on healthy lifestyles. Furthermore, students have a need to actively participate in decisions affecting their health and learning, such as the design and management of the school environment (Rising Holmström and Boström 2021).

The primary tasks of school nurses are to recognize and prevent adverse changes in health, identify health problems and take appropriate actions. Their true value lies in health promotion and disease prevention (NASH 2025; Arrue‐Gerra et al. 2024; Bergren 2017). School nurses are in a unique position to be associated with the health and well‐being of children, adolescents and their families.

Over the years, school nursing care has evolved to adapt to the changing health needs of students, which have become more complex. The number of students requiring increased care and those at risk due to increasing health problems is influenced by social determinants such as poverty and gaps in access to care. Additionally, school health reform in Hungary in 2006 was associated with the development of care (Odor 2010). With the establishment of the school nurse specialization, the care of school‐aged children, previously the responsibility of district nurses, was transferred in cities to full‐time school nurses. In villages, the health care of school‐aged children remained within the duties of district nurses due to smaller student numbers. Currently, one full‐time school nurse provides care for approximately 1000 students, which in cities often involves covering two schools per nurse.

In line with international practice, it placed greater emphasis on community‐based, coordinated care, while improving communication with families, healthcare providers and public health organizations (Nygård et al. 2025; Hustad et al. 2025). School nurses serve as a bridge between schools, families and community health resources (Dickson et al. 2025; Armas Junco et al. 2025). The functioning of the school health system is fundamentally based on partnership, trust and communication between schools and the health care system (Zhang et al. 2026).

In the 21st century, changes in students' health needs in healthcare have significantly influenced school nursing practice (Maughan et al. 2016). Hoekstra et al. also confirm in their study that the role of school nurses in health education has shifted over time from directly educating students to providing health promotion advice and support related to school health initiatives, including ensuring resources for teachers' tasks (Hoekstra et al. 2016). Today, there is an even greater demand for comprehensive public health tasks in schools due to the increase in diseases that cause health losses, which could be prevented through public health interventions. In our introduction, we aimed to provide a detailed presentation of the school nurse's responsibilities, focusing not only on conducting screening examinations but also on supporting students' physical and mental health issues and promoting healthy behaviours. Additionally, it is particularly important to understand the school nurse's activities from the students' perspective and how these activities help them develop and maintain health awareness in their daily lives.

### 2.1 The School Nurse (Health Visitor) System in Hungary

The health visitor care service in Hungary is part of a unique primary healthcare system, recognized as a Hungarikum, meaning it is specific to Hungary. As a cornerstone of primary care, the service primarily provides family and maternal protection. From the onset of pregnancy, health visitors monitor the expectant mother's health, establishing a close, personal and long‐term relationship with her.

One type of health visitor, the school nurse, also works with children aged 3–18 years. Every public educational institution has a school nurse responsible for the primary care of preschool and school‐aged children. Their duties include conducting health examinations every 2 years for students over 6 years of age, encompassing anthropometric measurements, assessment of physical, sexual, sensory and musculoskeletal development, as well as screening for psychological and behavioural issues. Additionally, they monitor personal hygiene, provide first aid, prepare students for medical examinations and coordinate vaccination‐related organizational tasks. They support students with chronic illnesses or behavioural disorders, participate in health education and maintain communication with parents through parent‐teacher meetings. Their work also extends to supporting career guidance and assisting students in their daily school life.

## 3 The Study

### 3.1 Aims and Objective

While previous studies have described school health services and the role of school nurses, the perceived effectiveness of school nurse activities from the students' perspective and its relationship with subjective health status remain underexplored, particularly in Central and Eastern Europe. This study addresses this gap by examining how Hungarian high school students evaluate the perceived effectiveness of school nurse services and how these evaluations are associated with key self‐reported health indicators.

Our research aimed to explore the perceived effectiveness of school nursing services from the student's perspective. We considered it important to understand both the quantitative indicators of the school nurse's health education and health promotion activities and their evaluation—specifically, their applicability according to students' responses. Additionally, we aimed to assess the association between school nursing services and health perceptions, as well as students' satisfaction with nursing activities. Our investigation also extended to examining students' subjective health status. We hypothesized that higher perceived effectiveness of school nursing services would be associated with better self‐reported mental health and vitality and with a lower level of subjective health complaints among students.

## 4 Methods

### 4.1 Design

A quantitative, cross‐sectional study was conducted in public educational institutions offering full‐time high school education in the West Transdanubian region of Hungary.

### 4.2 Sampling

We considered the inclusion criteria for the sample to be students enrolled in a four‐year daytime high school program, whose school health services were provided by full‐time school nurses and who had been attending the given school for at least 2 years. Students continuing their studies as private students were excluded.

The study applied a non‐probability (convenience) sampling approach at the school level, as participating institutions were selected based on accessibility and willingness to participate. Schools were approached through existing professional and institutional networks and those where approval from school leadership and local stakeholders could be most feasibly obtained were invited to participate. This approach likely favored schools with a higher perceived openness to health‐related research and may have introduced selection bias, limiting the representativeness of the sample. Within the selected schools, the primary sampling units were classes rather than individual students. Classes were selected using a systematic random sampling method (Pakai and Kívés 2013; Karamánné Pakai and Oláh 2015) and all students in the selected classes were invited to participate in the survey.

Data collection was conducted during school lessons. In total, 18 classes across 5 high schools were included, resulting in a final sample of 429 students. Data were collected using a group sampling procedure during school lessons, with students completing paper‐based questionnaires individually using the pen‐and‐paper method. The questionnaires were completed in classrooms and required approximately one class period (45 min), with an average completion time between 35 and 47 min. Although this duration is relatively long for adolescent respondents and may increase the risk of respondent fatigue, no major difficulties or disruptive behaviours were observed by the researchers or teachers during data collection and students generally completed the questionnaires without interruption. Students received standardized information about the study and researchers were available to answer questions during data collection.

### 4.3 Instruments

The measurement tools used in our research can be seen in Table 1.

**TABLE 1:** Variables measured and instruments used during the study.

Variable	Instrument	Items
QoL	SF‐36, EQ‐VAS	General health perception, vitality and mental health, current health status
Types of subjective psychological and somatic health complaints	HBSC (Health Behaviour in School‐Aged Children)	Fatigue/exhaustion, nervousness, feelings of apathy, headaches, sleep problems, abdominal pain, dizziness, back pain
Perceived effectiveness of school nursing	Self‐developed questionnaire	The nurse's activities on health education, the amount of health knowledge acquired, its applicability in daily life and student satisfaction with the nursing work
Demographic data		Residence, grade, age

First, a self‐developed questionnaire focusing on demographic data and the assessment of perceived effectiveness of school nursing was used.

Additionally, standardized questionnaires were also used. The 36‐item Short Form Health Survey (SF‐36) is widely used in both medical and other research fields to measure changes in health status. This questionnaire assesses subjective perceptions of health, capturing respondents' opinions on their health, with higher scores indicating better health. In our study, we utilized specific components of the SF‐36 questionnaire: general health perception, vitality and mental health (Ware et al. 1993).

For measuring health status, we also used the EQ‐VAS (EuroQol Visual Analogue Scale), where respondents indicate their current health status on a scale from perfect health (100 points) to the worst possible health (0 points), reflecting their position relative to these endpoints (Szende and Williams 2004).

To measure the occurrence of subjective health complaints among youths, we adapted questions from the HBSC (Health Behaviour in School‐Aged Children) questionnaire. This tool assesses the frequency or absence of nine subjective health complaints, including psychological and somatic symptoms (Németh and Költő 2016). The symptom scores can range between 9 and 45, where higher scores indicate greater frequency of subjective complaints.

The perceived effectiveness of school nurse care was assessed using a composite score derived from four closed‐ended items. All four items were measured on a four‐point Likert‐type scale, on which students assigned scores to different statements. During scale development, we first identified the main dimensions of perceived effectiveness based on the relevant literature, subjected the items to expert review and then formulated the final items accordingly. To assess validity, we conducted an exploratory factor analysis, which showed that the four items loaded on a single factor, supporting the unidimensional structure of the scale. In the factor analysis, the MSA value was high (0.879), the variables fitted well into the factor structure and Bartlett's test of sphericity was significant (*p* < 0.001), with a KMO value of 0.787, indicating sufficient correlations among the variables. After rotation, the extracted factor accounted for 64.41% of the total variance, exceeding the commonly used 60% criterion. The perceived effectiveness of school nurse care scale consisted of the following components: the association between the school nurse's activities and health‐related attitudes, the amount of health‐related knowledge received, its applicability in everyday life (quality indicator) and students' satisfaction with the school nurse's work. Responses to the four items were aggregated for each student, resulting in a composite score for each participant, which was treated as a continuous variable (between 4 and 16, where a higher total score indicated a more beneficial and productive nursing service). Higher total scores indicated higher perceived effectiveness. The items were summed to create a composite score, as Likert scales with adequate internal consistency are commonly treated as approximating interval‐level measurement in practice and their summed scores are widely used in quantitative analyses (Huh and Gim 2025; Carifio and Perla 2008). The reliability of the scale measuring the perceived effectiveness of school nurse care proved to be acceptable (Cronbach's alpha = 0.76). The questionnaire was pretested in a pilot study in one secondary school class (*n* = 28), during which we assessed item clarity and the functioning of the scale and used the feedback from this phase to finalize the instrument.

### 4.4 Data Analysis

In addition to descriptive statistics, mathematical statistical methods, including χ^2^ tests and correlation analysis were calculated. To assess normality (perceived effectiveness of school nurse care and symptom index), Kolmogorov–Smirnov and Shapiro–Wilk tests were applied; due to non‐normal distributions, non‐parametric tests (Spearman's rank correlation) were used. Correlation analyses were conducted using two‐tailed statistical tests with a significance level of 0.05. Following the bivariate analyses, we applied multivariable linear regression models to control for the effects of potential confounders. In these models, the health indicators were entered as dependent variables, perceived effectiveness of school nursing services as the independent variable and age, gender, place of residence and grade level were included as covariates. The data from the questionnaires were manually recorded using Microsoft Excel 365. Statistical analysis was performed using SPSS 27.0.

### 4.5 Ethics

Our research was conducted with careful consideration of ethical standards. The measurement tools were completed anonymously and did not include any information that could identify the students. We also informed the directors of the selected district centres about the research and additionally, we requested permission from the principals of the selected schools to conduct the data collection. We informed the parents of the affected students in writing about the research and requested their passive consent. Before the completion, the students were also informed about their participation in the research. Furthermore, the study complied with the Helsinki Declaration and data processing was managed in accordance with the European Union's general data protection regulation (GDPR 2016).

Institutional approval for conducting the study was obtained from the Szombathely District Education Centre (Nr. 18/05) and the Sárvár District Education Centre (Nr. 20/22). In addition, prior to data collection, the principals of the participating schools were informed about the study and their permission for conducting the survey was obtained.

Parents of the participating students received written information about the purpose, procedure and voluntary nature of the study and a passive consent procedure was used, whereby parents had the opportunity to refuse their child's participation. Before completing the questionnaire, students were also informed about the study and were reminded that participation was voluntary and that they could discontinue at any time.

## 5 Results

### 5.1 Sample Characteristics

In our research conducted in Vas and Zala counties in the West Transdanubian region, nearly half of the respondents (46.43%) were in the 10th grade, 39.29% in the 11th grade and 14.28% in the 12th grade. The mean age of the respondents was 16.87 years (SD: 0.82), with two‐thirds being female (67.79%). More than half lived in urban areas (31.46% in county seats and 27.46% in other towns) and two‐fifths resided in the same town as their school.

### 5.2 Assessing Subjective Health Status

With SF‐36, the average scores in the sample were: general health perception (M: 74.94 [SD: 17.31]), mental health (M: 68.33 [SD: 19.06]) and vitality (M: 50.47 [SD: 20.59]). The mean self‐reported health status on the 100‐point EQ‐VAS was 79.16 (SD: 16.5, max: 100, min: 10).

In addition to these measurements, we assessed the occurrence of subjective health complaints among youths using the HBSC (Health Behaviour in School‐Aged Children) research tool. Analysing subjective health complaints, fatigue/exhaustion and feelings of nervousness were the most frequently reported in the entire sample (daily: 24.11% and 13.17% respectively; 2–3 times per week: 22.77% and 31.03% respectively). Headaches (20.54%) and feelings of apathy (18.75%) were also noted. Among physical symptoms, headaches were predominant, affecting 14%–33% of the sample with varying frequencies. According to the ‘never’ responses, the least frequent complaints were dizziness (35.71%), sleep problems (26.12%), back pain (25.22%) and abdominal pain (13.62%). When examining complaints across nine areas, nearly half of the students (45.76%) experienced all nine symptoms over the past 6 months with varying frequencies. Complaints in eight areas were reported by 20.54% of the sample, seven types of symptoms by 14.29%, while the absence or non‐perception of four or fewer problems was minimal (7.37%). The mean symptom scale score was 20.67 (SD: 8.28).

### 5.3 Perceived Effectiveness of School Nurse Services

In evaluating the knowledge obtained from the school nurse, we noted that we did not specify areas of nursing activity, as knowledge expansion and changes related to health can occur not only during classroom health education but also through extracurricular activities. 89.04% of the students participated in the classroom‐based health promotion sessions conducted by the school nurse 1–2 times per year. Regarding extracurricular health promotion clubs and other events or activities led by the school nurse, the responses were much more varied. Around two‐fifths of the students took part in these, with the vast majority attending multiple times each semester. In addition to the encounters provided by screening examinations for all students, professionals working in school health services have the opportunity, during individual counselling—within the framework of consultation hours—to provide targeted, personalized care addressing all aspects of health, alleviate students' anxieties and fears and answer their questions and concerns. The school nurse's consultation hours were used by 23.88% of students (73.3% of them attended multiple times per semester, 26.7% once per semester), while the nurse proactively invited more students—35.48% of respondents—to participate in consultation hours or individual counselling (70.46% attended multiple times per semester, 29.54% once per semester).

**FIGURE 1:**
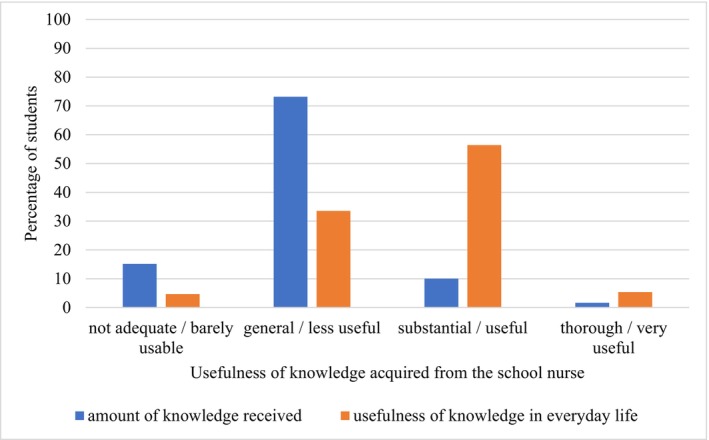
Perceived usefulness of knowledge acquired from the school nurse (*n* = 429).

For our first question, the quantitative results regarding the knowledge gained from the nurse were as follows: nearly three‐quarters of the students (73.19%) found the information provided to be sufficient, 15.15% thought it was not adequate, while slightly fewer found it substantial (10.2%) or thorough (1.63%). Based on students' responses to the following item assessing the quality indicator of knowledge received from the school nurse, three fifths of respondents felt that the health knowledge received from the nurse was very useful (5.36%) or useful (56.41%). However, it is notable that one‐third of the students (33.57%) considered it less and 4.66% found it barely applicable (Figure 1).

We considered it crucial to explore the relationship between the amount of health knowledge acquired and its practical applicability. Statistical analysis revealed a significant association between the expansion of health knowledge provided by school nurses and its practical application (χ^2^ = 15.89, *p* < 0.001). Cramér's V value indicating the strength of the association was 0.378, which, according to Cohen's guidelines, can be interpreted as a moderate association. The results suggest that as the amount of acquired health‐related knowledge increases, its practical applicability also tends to increase; however, the association cannot be considered deterministic. As the amount of knowledge increased, its perceived applicability also rose. Among those who considered the knowledge received from the school nurse sufficient, 65.2% found it useful or very useful. In contrast, the majority of those with a lot (93%) or substantial (96%) knowledge viewed it as useful or very useful. However, it is noteworthy that one‐third of those who deemed the knowledge sufficient found it less (33%) or barely applicable (1.2%). Future research should investigate areas where school nurse services might need renewal. Innovations in the content or methods could enhance the effectiveness of the health knowledge provided by school nurses to students.

**FIGURE 2:**
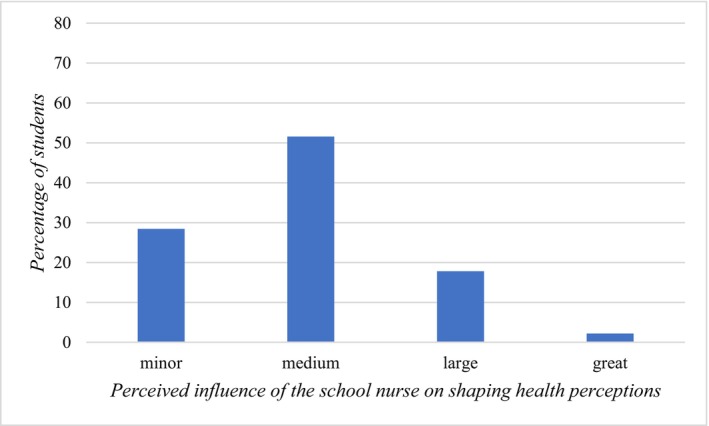
The association between the school nurse and health perception shaping (*n* = 320).

Our third question aimed to assess the effectiveness of school nursing work in changing health attitudes. Of the sample population (*n* = 320), 28.44% reported only slight, 17.81% experienced significant and 2.19% noted a very significant shift in their perception of health due to the activities of the school nurse (Figure 2).

As a result of our research, we examined the correlation between the quality indicators of school nursing services and their usage frequency and their association with health perception shaping. Based on the χ^2^ test, higher qualitative (χ^2^ = 114.71, *p* < 0.001) and quantitative (χ^2^ = 184.52, *p* < 0.001) evaluations of the utilization of school nurse services showed a statistically significant association with perceived health attitude–shaping related to the school nurse's activities within the school setting. The strength of the association, as indicated by Cramer's V, was 0.277 for the qualitative indicator and 0.389 for the quantitative indicator, which, according to Cohen's guidelines, can be interpreted as weak‐to‐moderate and moderate associations, respectively. These findings suggest that more frequent use of school nurse services, as well as more favourable qualitative evaluations, are associated with higher perceived levels of the school nurse's role in shaping students' health attitudes.

Our fourth question assessed the effectiveness of the school nursing service from the perspective of service satisfaction, using a rating scale from 1 to 4. Most students surveyed were satisfied with the nurse's activities, with nearly half (48.66%) expressing satisfaction, about a quarter (27.9%) being very satisfied and nearly the same number (22.32%) being somewhat satisfied.

We summed the scores from the four questions and considered a higher total score to reflect greater effectiveness in the school nurse's work. A higher level of perceived health‐related knowledge (provided by the school nurse), which is well utilized in daily life and is associated with health perception shaping, may be associated with more favourable health outcomes. Additionally, the level of satisfaction with the nursing service may reinforce or diminish the results from the areas examined. The maximum possible total score for the four questions was 16 points. The mean score of the 429 evaluable responses was 9.08 (SD: 2.19, max: 15, min: 4).

### 5.4 Relationship Between Perceived School Nurse Efficiency and Health Status

We determined the strength of the relationship between health indicators (SF‐36 dimensions and EQ‐VAS) and the perceived effectiveness of nursing services (scoring system as described in Subsection 4.3). The associations were examined using Spearman's rank correlation coefficient. The sample size (*N* = 429) provided sufficient statistical power, based on recommendations in the literature, to detect small effect sizes (*r* = 0.10–0.20) (Cohen 1988). The results indicated that the efficiency index showed a weak positive association with vitality (*r* = 0.19) and mental health (*r* = 0.214), as well as a weak negative association with the symptom index (r = −0.224) (Table 2). The proportion of explained variance ranged between 1% and 5%, which corresponds to a weak effect size according to Cohen's guidelines. To reduce the risk of Type I error due to multiple correlations, a Benjamini–Hochberg false discovery rate (FDR) correction was applied, after which statistically significant associations remained for mental health and the symptom index, however, the practical significance of these associations is limited due to the small effect size.

**TABLE 2:** Evaluation of the effectiveness of nurse services and the relationship between students' self‐assessed health status indicators based on Spearman's rank correlation calculation results (*N* = 429).

	*r* value	*p*
Vitality	*r* = 0.19	*p* = 0.015
Mental health	*r* = 0.214*	*p* = 0.026
General health perception	*r* = 0.067	*p* = 0.219
EQ‐VAS	*r* = 0.054	*p* = 0.251
Symptom index	*r* = −0.224*	*p* = 0.014

*Note: p* values were evaluated using the Benjamini–Hochberg false discovery rate (FDR) correction; associations marked with an asterisk remained statistically significant after correction.

* *p* < 0.05.

To control for the effects of potential confounding variables, we performed multivariable linear regression analyses in which age, gender, place of residence and grade level were included as covariates. The results showed that the perceived effectiveness of school nursing services was weakly but statistically significantly associated with mental health (*B* = 0.752; *β* = 0.104; *p* = 0.040) and showed a weak, negative association with the symptom index (*B* = −0.35; *β* = −0.107; *p* = 0.025), even after adjusting for the control variables. However, the explanatory power of the models remained low (mental health: *R* ^2^ = 0.206; symptom index: *R* ^2^ = 0.223). For vitality, the association between perceived effectiveness and the outcome did not reach statistical significance (*B* = 0.737; *β* = 0.089; *p* = 0.065; *R* ^2^ = 0.021). Similarly, no significant association was found for general health perceptions (*B* = 0.467; *β* = 0.067; *p* = 0.168; *R* ^2^ = 0.011). These findings suggest that perceived effectiveness of school nursing services is associated with certain health indicators; however, the magnitude of these associations is small and not detectable across all examined dimensions.

## 6 Discussion

The aim of our research was to assess the effectiveness of the school nurse's activities through the students' responses and to examine whether there is a detectable relationship between the students' subjective health status and the school nurse's service provision.

In terms of the health status of the respondent students, the general sense of health was considered adequate compared to standard values, but mental health was lower, which was accompanied by reduced vitality and symptoms of persistent fatigue, exhaustion, lack of enthusiasm and nervousness. According to our results, the general sense of health measured with the SF‐36 questionnaire was considered adequate compared to the standard values (M: 74.94), as the average of the Hungarian sample of the healthy population under 18 years was M: 74 (Czimbalmos et al. 1999). In the publication by Czimbalmos et al., the normal mean value for the healthy population under 18 years in Hungary for vitality and mental health was 79–79 (Czimbalmos et al. 1999), which showed a lower value in the current respondent students (mental health M: 68.33; vitality M: 50.47). However, based on the 2022 HBSC survey, it has been indicated that compared to the data collection in 2006, 2010, 2014 and 2018, the mental health results of school‐aged youth show a significantly decreasing trend (Németh and Költő 2016; Cosma et al. 2023). The momentary sense of health measured on the EQ‐VAS 100‐degree health thermometer was slightly lower in the examined sample (M: 79.16) than in the study by Szende and colleagues (M: 83.3) (Szende et al. 2014). Examining subjective health complaints, every second student noticed all the assessed symptoms over the past 6 months with varying temporal recurrence, but psychological problems were predominantly present; every second student complained of fatigue or exhaustion daily or several times a week and significant levels of lack of enthusiasm, irritability and nervousness also appeared. Our results and the international HBSC data were nearly identical (headache occurring several times a week: 20% vs. 20.54%, exhaustion: 25% vs. 22.77%; nervousness: 33% vs. 31.03%, respectively) (Cosma et al. 2023). The symptom index measured with the questions of the HBSC research did not significantly differ from the average of students in a similar age group (own research M: 20.67; HBSC M: 20.93) (Németh and Költő 2016). Based on these results, the subjective indicators of general health status did not significantly differ from national and international averages.

During the analysis of the data examined as components of the effectiveness of the school nurse's service provision, the students' responses indicated that the sufficient or greater amount of health‐related knowledge received from the school nurse and its applicability were associated with each other (*p* < 0.05). Three‐quarters of the students considered the acquired knowledge sufficient; however, one‐third rated it as less or barely usable. Three‐quarters of the surveyed youth reported that their health perspectives had developed in a more positive direction in connection with the school nurse's service. A more favourable change in students' health perspective was related to more frequent encounters with the school nurse (within the framework of school health education, screening examinations and consultation hours) and higher quality evaluations of her activities (*p* < 0.05). In the future, it may be important to incorporate practical, applicable knowledge in school nurse activities concerning key health topics, paired with appropriate methods, in order to better support the usefulness of this knowledge in students' everyday lives. We included satisfaction with the school nurse's activities in the concept of effectiveness, to which around three‐quarters of the students gave a positive response to a smaller or larger extent.

Examining the aggregated values of effectiveness, more productive service was associated with better mental health, higher vitality and reduced health complaints (*p* < 0.05). Overall, the results showed statistically significant associations between the perceived effectiveness of school nursing services and more favourable mental health and a lower symptom index; however, the magnitude of these associations was weak and the explained variance remained low. These findings indicate that perceived effectiveness is related to students' health indicators, but the practical significance of the relationships is limited and they should not be interpreted as strong or determinative. It is likely that many other factors not measured in this study (e.g., family background, broader psychosocial context) play a substantial role in shaping adolescents' health status.

The findings from the multivariable analyses similarly confirmed that the associations between perceived effectiveness of school nursing care and certain health indicators remained after controlling for relevant background variables, but again with small effect sizes. The significant relationships observed for mental health and the symptom index suggest that higher perceived effectiveness of school nursing care was associated with better psychological well‐being; yet these associations are modest in size and should be interpreted with caution rather than overemphasized.

Several studies also report associations between school nurses and the somatic and psychosocial health of students. These studies emphasize that school nurses play a key role not only in managing physical health problems but also in identifying and addressing mental health needs and supporting students' overall well‐being (Grispin et al. 2024; Flodin et al. 2025; McKay et al. 2025; Johansson et al. 2026). Our findings are in line with this international literature, as students who perceived school nursing services as more effective reported better mental health and fewer subjective health complaints, albeit with modest effect sizes.

One plausible explanation for these associations is that students who experience school nursing services as effective may receive more relevant health information, emotional support and guidance, which can contribute to higher health literacy and a greater sense of control and may thereby be related to better mental well‐being and fewer subjective complaints. In addition, a trusting relationship with the school nurse and repeated, low‐threshold contacts may encourage students to seek help at an earlier stage when problems arise, which could be associated with fewer psychological and somatic complaints.

The results highlighted the necessity of a continuous, school‐based service of personalized school nurse activities that consider individual needs for a more favourable change in students' health perspectives (Hoekstra et al. 2016). Increasing students' motivation to utilize school health services can also be supported by informing and involving teachers (Rising Holmström and Boström 2021; Sharma et al. 2025). Besides relationships with peers, trustful relationships established with school nurses and other staff enable the creation of a school environment where young people feel safe and supported. Future interventions should also focus on strategies aimed at strengthening students' in‐school relationships, fostering open communication, trust and commitment (McCabe et al. 2022; Hackett et al. 2026). It is important to familiarize students with the services of the school nurse and the possibilities of their utilization. Research results indicate that dialogue between students, the school and school health services can be beneficial in promoting both health and academic success of students (Kostenius 2023; Rising Holmström et al. 2015).

### 6.1 Limitations

While this study provides valuable insights, several limitations should be acknowledged. Firstly, the sample size, although adequate for detecting small effects in correlation and regression analyses, still limits the generalisability of the findings. Secondly, the sampling strategy was based on convenience at the school level and only schools that were accessible and willing to participate were included, which may have introduced selection bias and further limits the representativeness of the sample. As a result, the findings may not be fully generalisable to the broader school‐aged population. The cross‐sectional design does not allow for causal inferences; while the study examined associations between school nurse services and students' self‐reported health status, it cannot determine the actual impact of these services over time.

Additionally, the study relied exclusively on students' self‐reported data, which may be subject to perception and response biases. In line with this, the term effectiveness was operationalized as perceived effectiveness, meaning that the study measured students' evaluation of the usefulness and applicability of health‐related knowledge received from the school nurse, rather than objective health outcomes. Furthermore, the perceived effectiveness of school nursing services contributed only modestly to the variance in self‐rated health indicators and the effect sizes of the observed associations were small. This limits the practical significance of the findings and suggests that numerous other, unmeasured factors likely play a more important role in determining adolescents' health.

Furthermore, the study was conducted in a single region of Hungary and school health services, organizational cultures, and student populations may differ substantially in other regions or countries. Therefore, the findings should be interpreted as region‐specific and cannot be assumed to generalize to all settings. In addition, social desirability bias cannot be ruled out, as students may have tended to provide more favourable evaluations of school nurses' activities or their own health status in a school context, even though the questionnaires were completed anonymously. Also, the cross‐sectional design and the absence of longitudinal follow‐up do not allow any conclusions about temporal or causal relationships between perceived effectiveness of school nursing services and students' health indicators. Future longitudinal studies are needed to clarify how changes in school nursing practice may relate to changes in students' health over time.

To assess common method bias, Harman's single‐factor test was applied. The analysis showed that the first factor accounted for 48.35% of the total variance, which did not reach the commonly cited 50% threshold in the literature. Although Harman's single‐factor test did not indicate a dominant method bias, this procedure has limited sensitivity and thus the presence of bias cannot be completely ruled out. Moreover, the study relied exclusively on self‐reported data, which are particularly susceptible to various forms of response bias, such as social desirability, current mood and individual differences in the interpretation of items. It is especially important to note that both perceived effectiveness and health indicators were obtained from the same source, which increases the risk of common method bias and may artificially inflate the observed associations between variables. Accordingly, the results should be interpreted with particular caution. Finally, convenience sampling further limits the generalisability of the findings to other regions or student populations.

## 7 Conclusions

To support the perceived effectiveness of school nurse services, it may be important to focus on quality indicators and on ensuring that students receive continuous, practical and well‐usable health‐related knowledge embedded in everyday life and tailored to their needs (Jameson et al. 2022). School nurses appear to play a key role in delivering health education, identifying emerging health issues and supporting students' understanding and application of health knowledge. Our findings suggest that more favourable student perceptions of school nurse activities are associated with more positive health perceptions and fewer reported health complaints, although these associations are modest in size. These observations point to the potential value of targeted professional development for school nurses, with particular emphasis on health education strategies, communication skills and approaches to adapt interventions to students' needs.

## Author Contributions

Ilona Karácsony: conceptualization, statistical analysis, manuscript writing. Tímea Csákvári: manuscript writing, visualization. Annamária Pakai: conceptualization, manuscript writing, supervision.

## Funding

The authors have nothing to report.

## Disclosure

The authors affirm that the methods used in the data analyses are suitably applied to their data within their study design and context and the statistical findings have been implemented and interpreted correctly. The authors agree to take responsibility for ensuring that the choice of statistical approach is appropriate and is conducted and interpreted correctly as a condition to submit to the Journal.

## Ethics Statement

Institutional approval for conducting the study was obtained from the Szombathely District Education Centre (reference number: 18/05) and the Sárvár District Education Centre (reference number: 20/22). Our research was conducted with careful consideration of ethical standards. The measurement tools were completed anonymously and did not include any information that could identify the students. We also informed the directors of the selected district centres about the research and additionally, we requested permission from the principals of the selected schools to conduct the data collection. We informed the parents of the affected students in writing about the research and requested their passive consent. Before the completion, the students were also informed about their participation in the research. Furthermore, the study complied with the Helsinki Declaration and data processing was managed in accordance with the European Union's general data protection regulation.

## Consent

The authors have nothing to report.

## Conflicts of Interest

The authors declare no conflicts of interest.

## Data Availability

The data that support the findings of this study are available from the corresponding author upon reasonable request.
